# Stylistic Composition of Melodies Based on a Brain-Inspired Spiking Neural Network

**DOI:** 10.3389/fnsys.2021.639484

**Published:** 2021-03-11

**Authors:** Qian Liang, Yi Zeng

**Affiliations:** ^1^Research Center for Brain-Inspired Intelligence, Institute of Automation, Chinese Academy of Sciences, Beijing, China; ^2^School of Artificial Intelligence, University of Chinese Academy of Sciences, Beijing, China; ^3^National Laboratory of Pattern Recognition, Institute of Automation, Chinese Academy of Sciences, Beijing, China; ^4^Center for Excellence in Brain Science and Intelligence Technology, Chinese Academy of Sciences, Shanghai, China

**Keywords:** spiking neural network, spike-timing dependent plasticity, sequential memory, musical learning, melody composition

## Abstract

Current neural network based algorithmic composition methods are very different compared to human brain's composition process, while the biological plausibility of composition and generative models are essential for the future of Artificial Intelligence. To explore this problem, this paper presents a spiking neural network based on the inspiration from brain structures and musical information processing mechanisms at multiple scales. Unlike previous methods, our model has three novel characteristics: (1) Inspired by brain structures, multiple brain regions with different cognitive functions, including musical memory and knowledge learning, are simulated and cooperated to generate stylistic melodies. A hierarchical neural network is constructed to formulate musical knowledge. (2) Biologically plausible neural model is employed to construct the network and synaptic connections are modulated using spike-timing-dependent plasticity (STDP) learning rule. Besides, brain oscillation activities with different frequencies perform importantly during the learning and generating process. (3) Based on significant musical memory and knowledge learning, genre-based and composer-based melody composition can be achieved by different neural circuits, the experiments show that the model can compose melodies with different styles of composers or genres.

## 1. Introduction

Using artificial intelligence as a tool to analyze and create music pieces has been in practice for quite some time. The first melody generated by computer appeared in 1957, this work was developed by Mathews at Bell Laboratories (Briot et al., 2017). Like the model of the commonly cited example “The Illiac Suite,” early methods were mainly based on the complex rules system (Fernandez and Vico, 2014), combined with markov chain or L-system (Jones, 1981; Nelson, 1996; Lo and Lucas, 2006; Gale et al., 2013; Fernandez and Vico, 2014). In recent years, machine learning techniques, especially deep learning, have become a fast growing domain and used with increasing frequency for music analysis and creation. Researchers have developed diverse neural network architecture to generate music content, as autoencoder (Bretan et al., 2016; Sturm et al., 2016), Boltzmann machine (Lattner et al., 2016; Hadjeres et al., 2017), recurrent neural network (Mozer, 1994; Simon and Oore, 2017), long-short term memory (Eck and Schmidhuber, 2002; Johnson, 2017; Makris et al., 2017), generative adversarial networks (Dong et al., 2017; Wu et al., 2017). Some researchers have tried to study music using spiking neural network (SNN), for example, melody recognition (Fujii and Oozeki, 2006), musical memory (Liang et al., 2020), and interactive environment for human to create musical pieces (Kerlleñevich et al., 2011). However, the method on how to generate music pieces using a spiking neural network has not been explored. Based on the current situation, we try to propose a spiking neural network to create melodies with different styles.

Actually, music is part of human nature, it involves the human experience, social culture, domain specific knowledge and complex cognitive abilities. Listening and creating a music piece, engage personal memory, sensory perception, multimodal integration, action, emotion and etc. (Koelsch, 2012). Jules Combarieu once said, “music is the art of thinking with sounds.” How humans learn and make music may be quite different from the mathematical models mentioned above. When a musician writes a melody, his memory, emotion, musical knowledge and skills are involved rather than calculating mathematical numbers or probabilities. Actually, creative behavior is an extremely complex process for humans. Scientists have found that musical creative behaviors and improvisation need the participation of the memory system and knowledge experience (Dietrich, 2004). Working memory, long-term memory and auditory short-term memory have been found to be involved (Dietrich, 2004; Lu et al., 2015) and interacted with improvisation (Limb and Braun, 2008). Some neuroscientists have proposed one brain network, called default mode network (DMN), which is central to human creativity (Jung et al., 2013). The DMN contains several brain regions, including dorsomedial prefrontal cortex (DLPFC), ventromedial prefrontal cortex (VMPFC), lateral temporal cortex (LTC), inferior parietal lobule (IPL) and etc. (Bashwiner et al., 2016). This model provided a perspective on how human creativity might map to the brain. Experiments have implicated that the DMN is engaged in musical improvisation (Bengtsson et al., 2007; Limb and Braun, 2008; Bashwiner et al., 2016). Actually, dorsomedial prefrontal cortex is proved to be involved in memory system (Fuster, 2000a,b, 2001, 2002), Ventromedial prefrontal cortex(VMPFC) is relevant to conceptual knowledge representation and abstract category learning (Bowman and Zeithamova, 2018) and societal standards of a person's culture (Dietrich, 2004).

Inspired by the mechanisms of human creativity, we present a brain-inspired spiking neural network which is capable of creating melody based on musical memory and knowledge. Unlike traditional artificial intelligence methods, our model has the following innovative features:

- The model is composed of several collaborative subnetworks which are similar to corresponding regions in brain. Because of the importance of musical memory and musical knowledge during creative behaviors, this paper focus on building a complex sequential memory subsystem to store a large number of musical pieces, and a knowledge subsystem to learn musical knowledge.
- The structure of each subnetwork is different. A sequential memory network is composed of several layers to store the information of musical tracks, while a hierarchical structure is employed to learn the musical knowledge including the information of composers and genres.
- Different brain oscillation activities, including theta and gamma waves, play a key role during the learning process and composing process.
- Both excitatory and inhibitory neurons are employed in the network. The individual neural is simulated by the Izhikevich model. Excitatory and inhibitory synaptic connections with different transmission delays are also involved in the computation. The weights of synapses are updated by spike-timing dependent plasticity (STDP) learning rule.
- Melodies are generated with specific composer's styles or genres (such as baroque, classical, romantic, and modern genres). Different neural circuits are involved in these tasks. The musical corpus used in this paper is a public a classical MIDI dataset (Krueger, 2018), which is composed of a large number of MIDI files.

The remaining of this paper is organized as the following structure: section 2 describes the architecture and the central methods of the model. Section 3 shows the results of melody composition. Section 4 gives the summary and discusses the future work.

## 2. Models and Methods

### 2.1. Model Description

#### 2.1.1. Model Architecture

**Figure 1:**
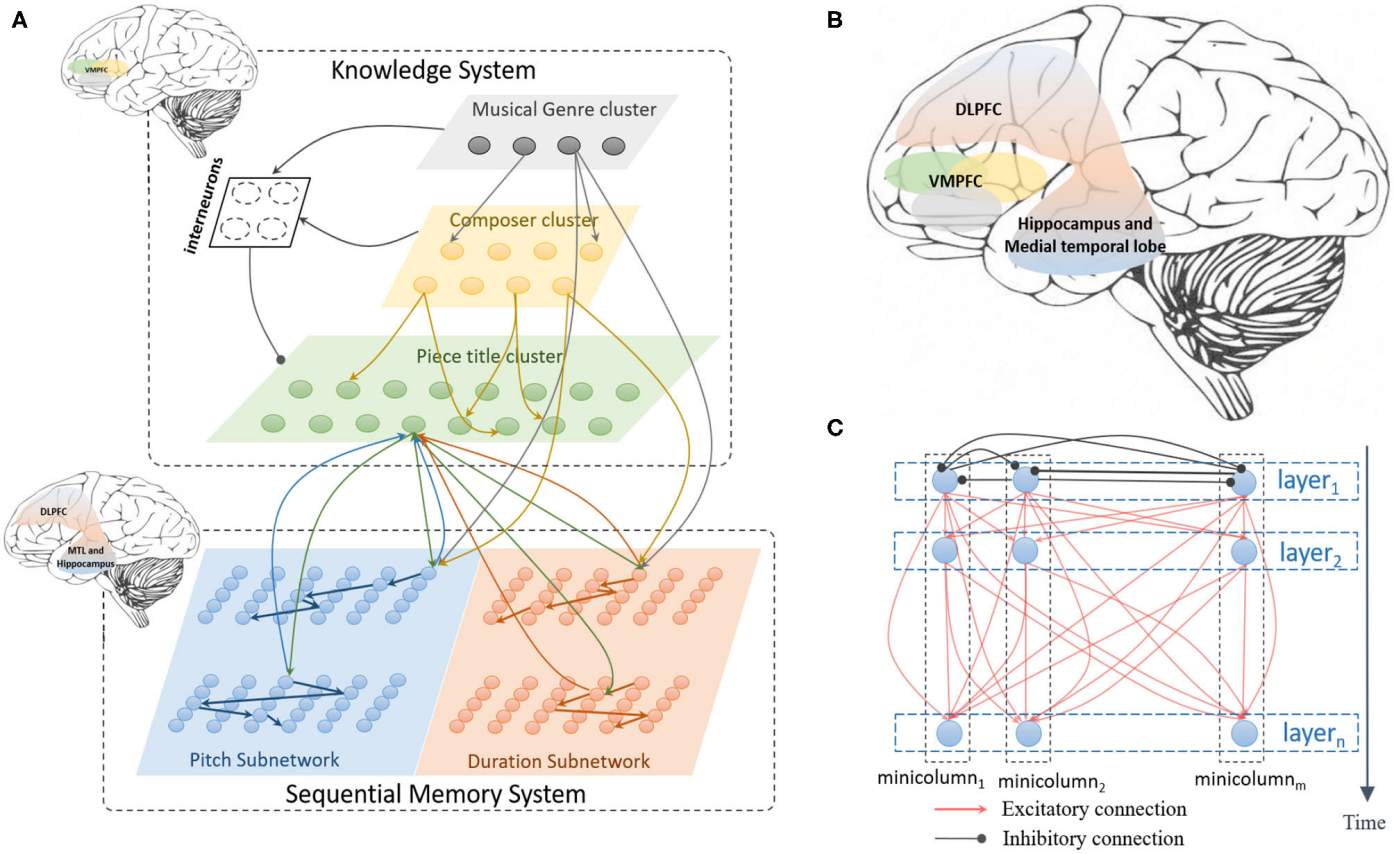
The architecture of the model, **(A)** describes the total architecture of the model, it contains knowledge and sequential memory subsystems, each subsystem is composed several areas which are inspired by relative brain regions marked in left top, **(B)** shows the main brain areas related in human memory and creativity, **(C)** draws the internal connection structure of the pitch and duration subnetworks in sequential memory system.

The architecture of the model is shown as Figure 1A. It mainly contains a sequential memory subsystem (Liang et al., 2020) and a knowledge subsystem. Musical memory is critical for creative behaviors (Dietrich, 2004), researchers have found that music is stored in different part of brain (Finke et al., 2012), including hippocampus, medial temporal lobe, dossolateral prefrontal cortex (Finke et al., 2012; Schaal et al., 2017) as shown in Figure 1B. However, the neural substrate on how these regions cooperate is not clear. Based on the existing neuroscience findings, this paper mainly aims to build a unified network to learn and store music pieces. As is shown in Figure 1C, the sequential memory system (Liang et al., 2020) is composed of pitch subnetwork and duration subnetwork. The pitch subnetwork (blue area) mainly encodes the pitch information and learns the ordered relationship between notes. The duration subnetwork (orange area) is responsible for representing the time interval between two adjacent notes. The building block of the pitch and duration subnetworks is non-overlapping functional minicolumns which is composed of numerous neurons. A group of neurons in the horizontal direction is called a layer. Neurons in the same layer are fully connected by inhibitory synapses, this connection mechanism helps active neurons to be more competitive at a given time. Synapses between neurons in different layers are excitatory, which express the ordered relationship and contextual information. Transmission delays of excitatory synapses depend on the length of the connection between two neurons, while inhibitory synapses have no transmission delays.

The knowledge subsystem is designed as a hierarchical structure to learn the information of composers and genres. As is shown in Figure 1A, the first layer (the gray area) is built to learn the genre of classical music pieces, such as the Baroque, Classical, Romantic, and Modern genres. The second layer (yellow area) is responsible for encoding the names of famous composers. The last layer (green area) represents the titles of musical pieces. Connections between these layers are dynamically generated and updated. Furthermore, neurons in the upper layers project to those in lower layers. Interneuron cluster, composed of numerous inhibitory neurons, receives signals from the neurons of the first (genre) and the second (composer) cluster, and projects the inhibitory connections to the third (piece title) layer. However, there are no connections between neurons in each cluster in this subsystem.

Connections from the knowledge clusters to the memory subnetworks are dynamically generated and updated. As Figure 1A shows that neurons in genre and composer clusters project the excitatory synapses to neurons in the memory subsystem. However, both feedforward and feedback connections are generated between neurons in the title cluster and memory subnetworks. It should be noted that the weights of connections between interneurons and clusters in the knowledge system are set to be a fixed value and not changed during the learning process.

#### 2.1.2. Neuron Model

Considering the balance of biological plausibility and the computational efficiency, this paper applies the Izhikevich neuron model (Izhikevich, 2003) to build the spiking neural network, which can be described as Equations (1)–(3), where *v* and *u* represent the membrane potential and a membrane recovery variable of a neuron, respectively. *a*, *b*, *c*, and *d* are parameters that control the model to fire with different spiking patterns. *I* is the input current, which carries information from external stimuli and synaptic currents from other neurons. The neuron emits a spike when the membrane potential *v* exceeds the peak value (30 mV), and *u* and *v* are reset to the initial values.

(1) $$\begin{array}{l} \frac{dv}{dt}=\eta \left(v,u\right)+I \end{array}$$

(2) $$\begin{array}{l} \frac{du}{dt}=a\left(bv-u\right) \end{array}$$

(3) $$\begin{array}{l} \eta \left(v,u\right)=0.04v^{2}+5v+140-u \end{array}$$

(4) $$\begin{array}{l} \text{if }v\geq 30\text{ mV, then }\{\begin{array}{l} v\leftarrow c\\ u\leftarrow u+d \end{array} \end{array}$$

The Izhikevich model is capable of simulating multiple spiking patterns of biological neurons with different morphologies and types. The most typical type of excitatory neurons in mammalian cortex is the regular spiking (RS) pattern with the parameters *a* = 0.01, *b* = 0.2, *c* = −65, and *d* = 8. While the inhibitory cells always exhibit fast spiking (FS) pattern with the parameters *a* = 0.1, *b* = 0.2, *c* = −65, and *d* = 2.

#### 2.1.3. STDP Learning Rule

Spike-timing dependent plasticity (STDP) is believed as one of the most important mechanism for brain learning and memory (Gerstner et al., 1996; Bell et al., 1997; Bi and Poo, 1999; Poo, 2008). Synapses between neurons are enhanced when the postsynaptic neuron fires a short time after the presynaptic neuron fires. Otherwise, synapses are depressed. STDP learning rule is described as the Equation (5)

(5) $$\begin{array}{l} \Delta w_{ij}=\{\begin{array}{cc} A_{+}e^{\Delta t/\tau_{+}}, & \Delta t<0\\ -A_{-}e^{-\Delta t/\tau_{-}}, & \Delta t>0 \end{array} \end{array}$$

where, the *w*_*ij*_ is the weight of the synapse from neuron *j* to neuron *i*, *A*_+_, *A*_−_ are the learning parameters, and τ_+_, τ_−_ are time constants. Δ*t* expresses the time difference between the presynaptic neuron *j* and postsynaptic neuron *i*.

#### 2.1.4. Oscillation Activities

Human brain oscillatory activities have been proved to be related to cognitive processes. Brain rhythms of different frequency may occur in different brain networks (Sauseng and Klimesch, 2008). Until now, Delta (0–4 Hz), theta(4–8 Hz), alpha(8–13 Hz), beta (13–30 Hz), and gamma (30–80 Hz) waves are found in human brain. However, researchers have found that theta oscillations exist in human cortex and hippocampus (Kahana et al., 2001). This wave are very important in memory encoding (Sederberg et al., 2003), information held (Jensen and Tesche, 2002) memory and episodic memory (Klimesch et al., 2001a,b). Researchers also have found that the gamma waves arise from excitatory and inhibitory circuits in cortex (Llinas et al., 1991). However, based on the studies mentioned in section 1, the brain areas involved in human creative activities, especially musical creativity mainly locate in cortex. Hence, we hypothesize that neurons in this paper exhibit theta and gamma waves during the memory and the creative process, respectively. It should be noted that internuerons exhibit fast spiking patterns, these neurons are always active at gamma frequency. Hence, we hypothesize that interneurons have no contributes to the memory process in this paper.

### 2.2. Model Implementation

#### 2.2.1. Information Encoding

**Figure 2:**
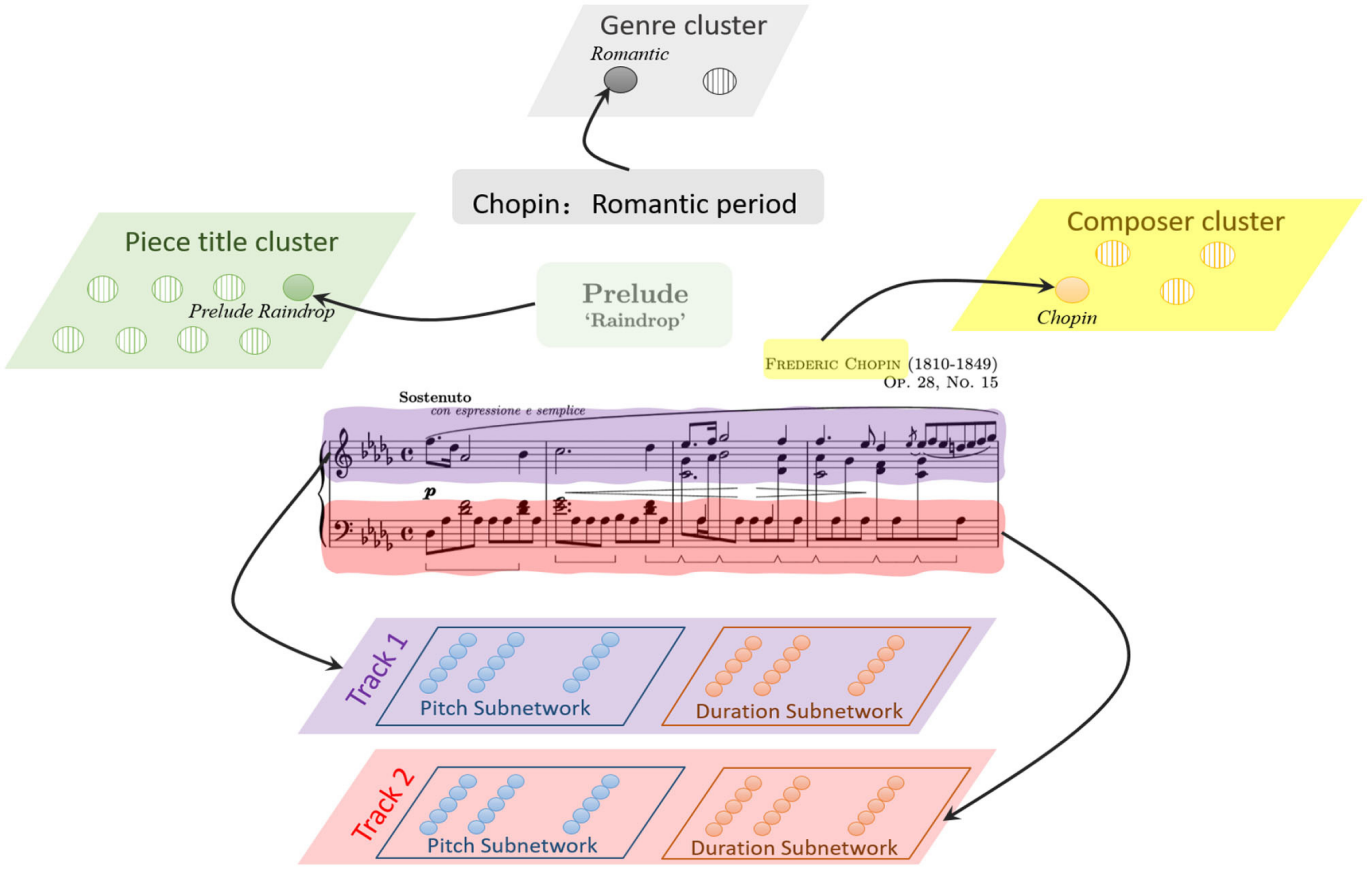
The representation of a music piece in our model, the basic information, including the genre, composer and title are encoded by corresponding cluster in knowledge system, while the music notes are encoded in sequential memory system.

Encoding is a critical but difficult task, and it should be solved first. As is shown in Figure 2, a musical piece includes the basic information of genre, composer, title, and sequential ordered notes. It's been found that neurons have specific selectivity in different brain regions. This property makes a neuron only respond to its preference. For example, evidence has shown that neurons in the cochlear nucleus are sensible for different frequencies of pitches (McDermott and Oxenham, 2008; Oxenham, 2012). Orientation columns in the visual cortex are excited about their preferred directions (Hubel and Wiesel, 1968). To explain the following parts clearly, the involved notations and parameters are listed in Table 1.

**Table 1:** The important notation description.

**Notations**	**Description**
*n*(*p, d*)	A note is represented as a tuple in which contains pitch and duration
$n_{ij}^{\left(P\right)}$	The neuron in the layer *i* of the column *j* in pitch subnetwork
$n_{ij}^{\left(D\right)}$	The neuron in the layer *i* of the column *j* in duration subnetwork
$v_{ij}^{\left(P\right)},u_{ij}^{\left(P\right)}$	The membrane potential and recovery variable of the neuron in layer *i* of column *j* in the pitch subnetwork
$v_{ij}^{\left(D\right)},u_{ij}^{\left(D\right)}$	The membrane potential and recovery variable of the neuron in layer *i* of column *j* in the duration subnetwork
$v_{i}^{\left(T\right)},u_{j}^{\left(T\right)}$	The membrane potential and recovery variable of the neuron *i* in the title cluster
$v_{i}^{\left(C\right)},u_{j}^{\left(C\right)}$	The membrane potential and recovery variable of the neuron *i* in the composer cluster
$v_{i}^{\left(G\right)},u_{j}^{\left(G\right)}$	The membrane potential and recovery variable of the neuron *i* in the genre cluster
$w_{ij}^{\left(C,G\right)}$	The weight between the post-synaptic neuron *i* in the composer cluster and the pre-synaptic neuron *j* in the genre cluster
$w_{ij}^{\left(T,G\right)}$	The weight between the post-synaptic neuron *i* in the title cluster and the pre-synaptic neuron *j* in the genre cluster
$w_{ij}^{\left(T,C\right)}$	The weight between the post-synaptic neuron *i* in the title cluster and the pre-synaptic neuron *j* in the composer cluster
$w_{ij}^{\left(P,mn\right)}$	The weight between the post-synaptic neuron $n_{ij}^{\left(P\right)}$ and the pre-synaptic neuron $n_{mn}^{\left(P\right)}$ in the pitch submetwork
$w_{ij}^{\left(D,mn\right)}$	The weight between the post-synaptic neuron $n_{ij}^{\left(D\right)}$ and the pre-synaptic neuron $n_{mn}^{\left(D\right)}$ in the duration submetwork
$w_{ij,k}^{\left(P,G\right)}$	The weight between the post-synaptic neuron $n_{ij}^{\left(P\right)}$ and the pre-synaptic neuron *k* in genre cluster
$w_{ij,k}^{\left(P,C\right)}$	The weight between the post-synaptic neuron $n_{ij}^{\left(P\right)}$ and the pre-synaptic neuron *k* in composer cluster
$w_{ij,k}^{\left(P,T\right)}$	The weight between the post-synaptic neuron $n_{ij}^{\left(P\right)}$ and the pre-synaptic neuron *k* in title cluster
$w_{ij,k}^{\left(D,G\right)}$	The weight between the post-synaptic neuron $n_{ij}^{\left(D\right)}$ and the pre synaptic neuron *k* in genre cluster
$w_{ij,k}^{\left(D,C\right)}$	The weight between the post-synaptic neuron $n_{ij}^{\left(D\right)}$ and the pre-synaptic neuron *k* in composer cluster
$w_{ij,k}^{\left(D,T\right)}$	The weight between the post-synaptic neuron $n_{ij}^{\left(D\right)}$ and the pre-synaptic neuron *k* in title cluster
*I*_*ext*_	The input current from external stimulation

##### 2.2.1.1. Notes Encoding

**Figure 3:**
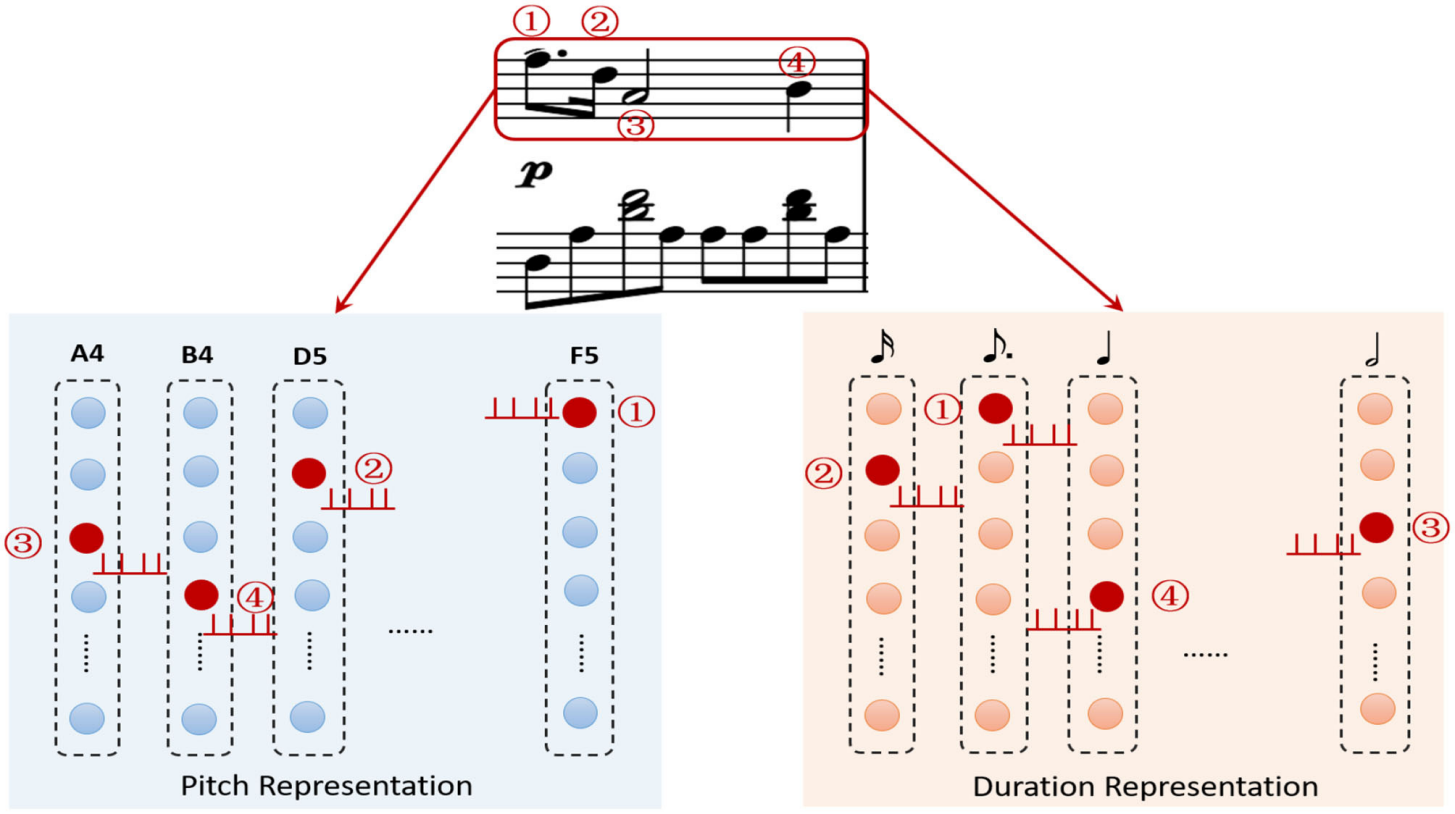
The description of note encoding, the pitch of a note is represented by the minicolumn which prefers it. The duration subnetwork encodes the duration of a note in the same way. The ordered relationships of the notes are stored by the layers of these subnetworks.

A musical piece contains multiple tracks, a track is composed a sequence of ordered notes. One note has two key attributes, pitch and duration. The most important task is how to represent these features. Unlike mathematical methods, neuroscientific researchers have found that the neurons in brain auditory cortex have their preferred pitch (Merchant et al., 2013), and neural populations in medial premotor cortex are sensitive for different time intervals in hundreds of milliseconds (Kalat, 2015). Moreover, numerous minicolumns composed of hundreds of neurons with their specific preferences are distributed widely in the brain cortex. Based on these mechanisms, we construct a pitch subnetwork and a duration subnetwork to represent these two attributes of the ordered notes. Since the MIDI protocol defines 0–127 digital numbers to define 128 pitches (e.g., 60 for middle C), the pitch subnetwork contains 128 functional minicolumns to represent them. Here, we set the pitch index as the preference for each neuron. Neurons in a minicolumn have the same preference as shown in Figure 3. When the external stimulation (pitch index) comes, a neuron transforms it to the input current using the Gaussian filter. If the neuron prefers the input pitch, it exhibits regular spiking activities. The current caused by external stimulation of neurons in pitch and duration subnetworks are calculated by Equation (6).

(6) $$\begin{array}{l} I_{ij\text{\_}ext}^{\left(P\right)}=\lambda_{1}\frac{1}{\sigma_{j}^{\left(P\right)}}e^{\frac{-{\left(x-\mu_{j}^{\left(P\right)}\right)}^{2}}{{\left(\sigma_{j}^{\left(P\right)}\right)}^{2}}} \end{array}$$

where *x* is the external stimulation, in other words, it is the input pitch. $\mu_{j}^{\left(P\right)}$ and $\sigma_{j}^{\left(P\right)}$ are the mean and variance of the neuron. Actually, $\mu_{j}^{\left(P\right)}$ is the preference of the neuron in the *i*th column. λ_1_ is the constant to make the neuron fire in theta waves.

Similarly, neurons in the duration subnetwork receive the time interval as the external stimulation and fire if they preferred the external information. Since the MIDI file commonly denotes 480 ticks for a crotchet, we create 64 minicolumns to express from a demisemiquaver (about 60 ticks) to two semibreves (3,960 ticks). Input current is described as Equation (7)

(7) $$\begin{array}{l} I_{ij\text{\_}ext}^{\left(D\right)}=\lambda_{2}\frac{1}{\sigma_{j}^{\left(D\right)}}e^{\frac{{-(x-\mu_{j}^{\left(D\right))})}^{2}}{{\left(\sigma_{j}^{\left(D\right)}\right)}^{2}}} \end{array}$$

where, *x* is the duration of input note, $\mu_{j}^{\left(D\right)}$ and $\sigma_{j}^{\left(D\right)}$ is the mean and variance, respectively. λ_2_ is the constant to make the neuron fire with theta frequency.

As the example shown in Figure 3, four ordered notes in the first track have been encoded by these two subnetworks. The first note triggers the neurons (red circles) encoding “F5” and “dotted quaver” in pitch and duration subnetwork to launch spikes, respectively. Then the following notes cause the corresponding neurons (red circles) to fire in order.

##### 2.2.1.2. Musical Knowledge Encoding

The basic information, including the genre, composer and title, can be seen as the corresponding knowledge of a piece, and represented by genre cluster, composer cluster and piece title cluster, respectively. Each cluster contains numerous neurons. Each neuron in the genre cluster stands for a genre (such as Baroque), which means that this neuron set the “Baroque” as its preference. Similarly, each neuron in the composer or title cluster also represents a composer name (“Chopin”) or a piece title (“Prelude Raindrop”). Neurons in these clusters are simulated by Izhikevich model with RS pattern as Equation (8), the input current *I*_*ext*_ is calculated as the Equation (8),

(8) $$\begin{array}{l} I_{ext}=\{\begin{array}{l} 5,\text{if external stimulation matches the neuron preference}\\ 0,\text{otherwise} \end{array} \end{array}$$

where, as the example of Figure 3, the external stimulation is “Romantic,” “Chopin,” and “Prelude Raindrop” for the neuron in genre, composer and piece title cluster, respectively.

#### 2.2.2. Music Learning

**Figure 4:**
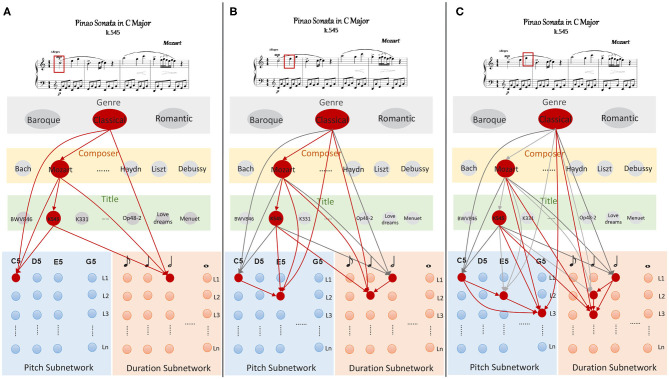
The storing process of the work “K545” written by Mozart, **(A)** describes the learning process of the first note marked by a red rectangle, the second and the third notes are leaned and the corresponding synapses are potentiated using STDP in **(B,C)**.

The learning task is the foundation of melody composition. It is a complicated process in which musical knowledge and pieces should be memorized and the relationship between them needs to be established. In the beginning, neurons are at rest, and the weights of excitatory synapses are set to zero. In order to describe the model clearly, we take the first measure of Mozart's work, “Sonata No. 16 in C Major (K545)” as an example to explain the learning procedure, and it's well-known that Mozart is a representative and great musician in the western classical period. As is illustrated in Figure 4,

- **Step1**
  The knowledge clusters allocate neurons to express the strings of “Classical,” “Mozart,” and “K545,” respectively. Based on the section 2.2.1, these neurons emit spikes simultaneously using Equation (8). Meanwhile, the first coming note triggers the neurons which preferred “C5” and a semibreve (960 ticks) to fire in pitch and duration subnetworks. These two neurons inhibit other neurons in the same layer in order to make themselves more competitive. Note that neurons in knowledge clusters are synchronous in theta waves, the synapses (red arrows in Figure 4A) from neurons in knowledge cluster to those in memory clusters are dynamically generated and strengthened using STDP learning rule as Equation (5). Similarly, synapses are generated and modulated between neurons from superior to inferior layers in knowledge clusters. Here, the time window for which each neuron continues to fire is set to 1 s, after which the membrane potential decays to 0 mV.
- **Step2**
  Similarly, the second note makes the neuron which prefers “E5” in pitch subnetwork fire, and the one in duration subnetwork which is sensible for the time length of a crotchet (480 ticks) also becomes excitatory. Note that neurons in knowledge clusters remain active throughout the learning process. It's reasonable that a person always keeps the name of a musical piece in his mind when he learns this piece. At this step, new connections are generated and potentiated using STDP, as shown in Figure 4B.
- **Step3**
  Neurons representing for “G5” and 480 ticks launch spikes with theta frequencies. New excitatory synapses are generated and updated between knowledge clusters to memory subnetworks. In addition, neurons which prefer “G5” and crochet in *L*_3_ also exactly receive the spikes from those in *L*_1_ due to the synaptic transmission delays (Liang et al., 2020).

Based on the learning process described above, membrane potentials of neurons in knowledge cluster are updated by the Equations (9) to (11).

(9) $$\begin{array}{l} v_{i}^{\left(G\right)}=\eta \left(v_{i}^{\left(G\right)},u_{i}^{\left(G\right)}\right)+I_{ext} \end{array}$$

(10) $$\begin{array}{l} v_{i}^{\left(C\right)}=\eta \left(v_{i}^{\left(C\right)},u_{i}^{\left(C\right)}\right)+\epsilon_{\theta }\left(I_{ext}+\underset{j}{\sum }w_{ij}^{\left(C,G\right)}\right) \end{array}$$

(11) $$\begin{array}{l} v_{i}^{\left(T\right)}=\eta \left(v_{i}^{\left(T\right)},u_{i}^{\left(T\right)}\right)+\epsilon_{\theta }\left(I_{ext}+\underset{j}{\sum }w_{ij}^{\left(T,C\right)}+\underset{j}{\sum }w_{ij}^{\left(T,G\right)}\right) \end{array}$$

where, ϵ_θ_(·) is a linear normalized function to control the current to a suitable value, since neurons exhibit the theta waves in this learning process. Neurons in pitch and duration subnetworks are updated by Equation (12) to equation 13.

(12) $$\begin{array}{l} v_{ij}^{\left(P\right)}=\eta \left(v_{ij}^{\left(P\right)},u_{ij}^{\left(P\right)}\right)+\epsilon_{\theta }(I_{ext}+\underset{m,n}{\sum }w_{ij}^{\left(P,mn\right)}\\ \;+\underset{k}{\sum }w_{ij,k}^{\left(P,T\right)}\underset{k}{\sum }w_{ij,k}^{\left(P,C\right)}+\underset{k}{\sum }w_{ij,k}^{\left(P,G\right)}) \end{array}$$

(13) $$\begin{array}{l} v_{ij}^{\left(D\right)}=\eta \left(v_{ij}^{\left(D\right)},u_{ij}^{\left(D\right)}\right)+\epsilon_{\theta }(I_{ext}+\underset{m,n}{\sum }w_{ij}^{\left(D,mn\right)}\\ \;+\underset{k}{\sum }w_{ij,k}^{\left(D,T\right)}\underset{k}{\sum }w_{ij,k}^{\left(D,C\right)}+\underset{k}{\sum }w_{ij,k}^{\left(D,G\right)}) \end{array}$$

where, all the notations involved have been described in Table 1.

**Figure 5:**
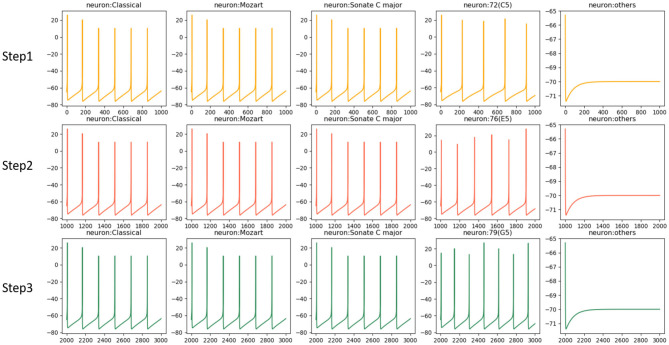
The neural oscillations in the learning process. Neurons preferred external stimulations including “Classical,” “Mozart,” “Sonate C Major” and related pitches have theta activities. Other neurons stay silent since they are not triggered.

**Figure 6:**
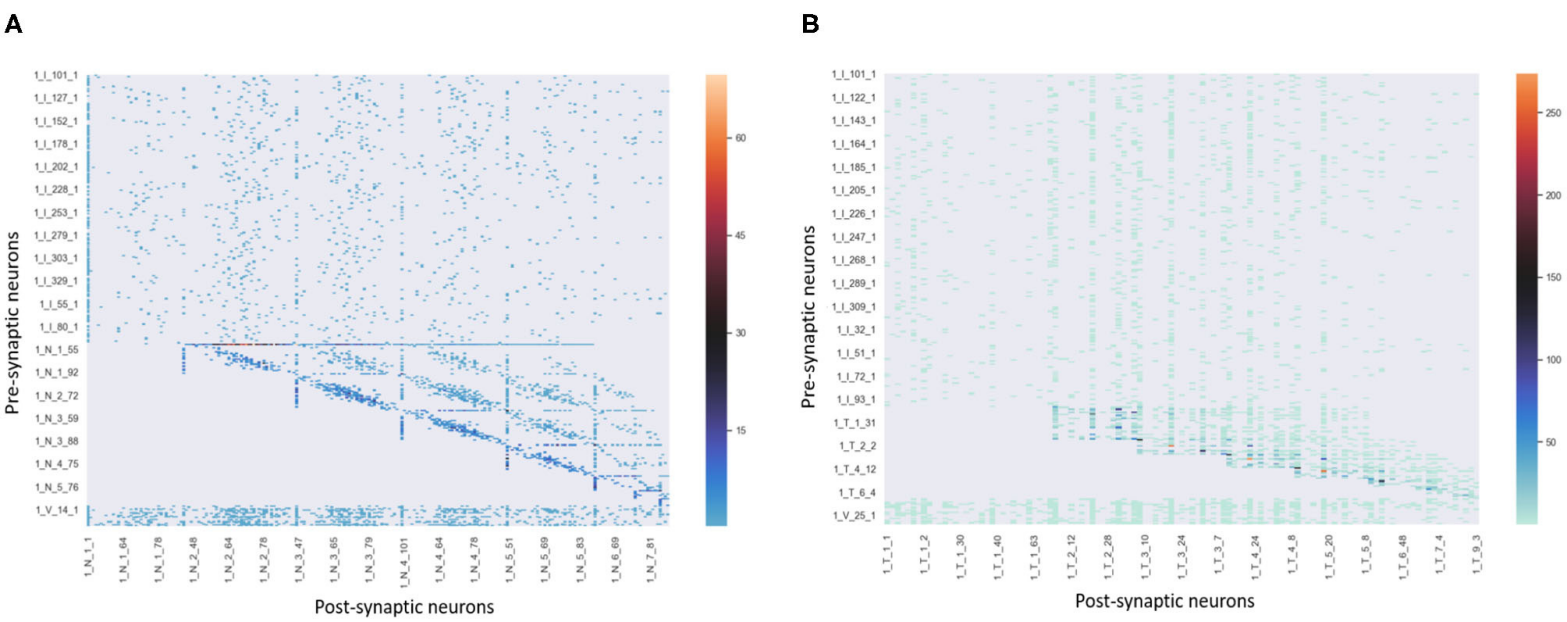
The synaptic strengths between neurons in pitch and duration subnetworks of track 1 during learning process.

Figure 5 shows the activities of related neurons mentioned above in this process. The neurons which prefer “Classical,” “Mozart,” and “Sonate C Major” in knowledge clusters are stimulated and exhibit theta waves simultaneously. Neurons in the pitch subnetwork are triggered orderly and also exhibit theta waves. The graph mainly shows the activated neurons' activities during the time windows 1 (s), after which the membrane potentials will decay to 0 mV. For simplicity, the figure only shows the results of neurons in the pitch subnetwork, the neurons in the duration subnetwork have similar activities. Besides, the synaptic weights between neurons in pitch and duration subnetwork are shown in Figure 6. Since the synapses are generated and updated dynamically during the learning process, the network is not full-connected. There are 331 musical works in our dataset, the graph only draws the results of the weight after the network learns 10 notes in track 1 of each piece for simplicity.

#### 2.2.3. Music Retrieval

After the music encoding and memorizing process, how the model retrieves this musical information is a significant problem. Actually, the retrieval process is a decoding process in this model. We have discussed the retrieval problem from two aspects, goal-based retrieval and episodic retrieval. The goal-based retrieval means that the model can remember all the sequential notes given a musical piece title. The episodic retrieval is that given an episode of a musical piece, the model can accurately recall the whole piece. Since this paper mainly focuses on melody composition, the detail of decoding process is described and emphasized in our previous work (Liang et al., 2020).

### 2.3. Melody Composition

If we listen to music from different periods, we can hear wide variations between them. The baroque, classical and romantic genres have their special styles. Similarly, the works of other musicians also have strong personal characteristics. For example, Johann Sebastian Bach, the father of western music, is a great musician in the baroque era. His works are mainly composed of religious music and polyphonic music, which are well-conceived and full of philosophy and logic. However, as a representative of the romantic ear, Fryderyk Franciszek Chopin's compositions demonstrate many characteristics of the romantic period. He is always trying to express his emotions, thoughts, and feelings in his music. Based on these inspirations, this paper mainly focuses on how to create melodies according to genre or composer characteristics. In this process, all the neurons exhibit gamma oscillations. Meanwhile, an initial notes array of non-fixed length, and the melody length should be given at first.

#### 2.3.1. Genre-Based Composition

**Figure 7:**
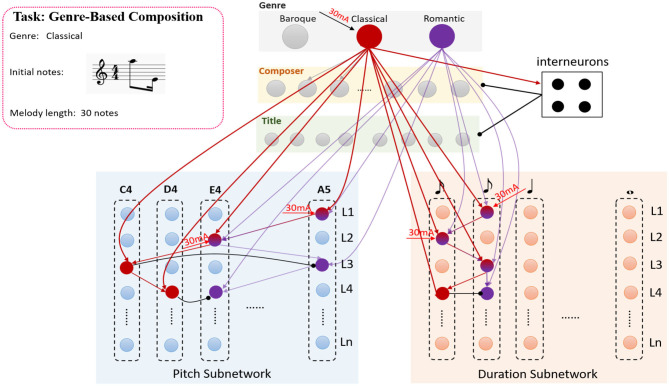
The process of the genre-based composition, the example in this graph is to create a melody with a classical genre, the genre cluster-interneurons-memory system circuits are active in this process.

Since the relationships between genre cluster and memory system have been established by learning a large number of musical pieces with various styles, the genre-based composition can be achieved by the collaboration of these subnetworks. The neural circuits of genre cluster-interneurons-memory system are involved in this process. A simple example is shown in Figure 7. Suppose that the model has stored two pieces which belong to classical and romantic genres, respectively. All the synapses have been trained in the learning process but have been drawn simplified in this figure. The task is to create a melody with classical style which contains 30 notes and begins with the notes *n*(*A*5, 240) and *n*(*E*4, 120), The time length of a crotchet is set to 480 ticks. In the beginning, neurons in genre cluster receive the external stimulation “classical” and transform this simulation to the input current, the neuron who prefers “classical” fires in gamma wave. Neurons in the genre cluster can be updated by Equation (9). However, the current *I*_*ext*_ is set to be 30(mA) to keep neurons fire in gamma frequencies if the external stimulation is matched with the neural preference. Then each neuron in knowledge clusters can be updated by Equations (14) to (16).

(14) $$\begin{array}{l} v_{i}^{\left(inh\right)}=\eta \left(v_{i}^{\left(inh\right)},u_{i}^{\left(inh\right)}\right)+\epsilon_{\gamma }\left(\underset{j}{\sum }w_{ij}^{\left(inh,G\right)}\right) \end{array}$$

where, $v_{i}^{\left(inh\right)}$ and $u_{i}^{\left(inh\right)}$ are the membrane potential and recovery variable of the interneuron *i*, $w_{ij}^{\left(inh,G\right)}$ is the weight of synapse from the genre neuron *j* to the interneuron *i*. ϵ_γ_(·) is also a normalization function to control the neuron fire with gamma frequency.

(15) $$\begin{array}{l} v_{i}^{\left(C\right)}=\eta \left(v_{i}^{\left(C\right)},u_{i}^{\left(C\right)}\right)+\epsilon_{\gamma }\left(\underset{j}{\sum }w_{ij}^{\left(C,G\right)}+\underset{j}{\sum }w_{ij}^{\left(C,inh\right)}\right) \end{array}$$

where, $\underset{j}{\sum }w_{ij}^{\left(C,inh\right)}$ is the total inhibitory inputs from interneurons to the neuron *i* in composer cluster.

(16) $$\begin{array}{l} v_{i}^{\left(T\right)}=\eta \left(v_{i}^{\left(T\right)},u_{i}^{\left(T\right)}\right)+\epsilon_{\gamma }\left(\underset{j}{\sum }w_{ij}^{\left(T,G\right)}+\underset{j}{\sum }w_{ij}^{\left(T,inh\right)}\right) \end{array}$$

Where, the $\underset{j}{\sum }w_{ij}^{\left(T,inh\right)}$ is the total inputs to inhibit the neuron to fire in title cluster.

As is shown in Figure 7, the process can be described as the following steps.

- **Step1**
  the first given note *n*(*A*5, 240) make the corresponding neurons in *L*_1_ of the pitch and duration subnetwork to fire with gamma frequencies.
- **Step2**
  Similarly, the neurons in *L*_2_ of pitch and duration subnetworks are stimulated by the second note *n*(*E*4, 120) and exhibit spikes.
- **Step3**
  Since the synapses in the model have been trained, both neurons representing “C4” (red circle) and “A5” (purple circle) in *L*_3_ of pitch subnetwork can be activated. However, the neuron encoding “C4” also receives the stimulation from the one which represents “classical” in the genre cluster. Hence, this neuron (red circle) emits spikes firstly and inhibits the one which encodes “A5” in the same layer because of inhibitory connections. The neuron which represents a quaver in the duration is triggered at the same time. These neurons can be updated by the Equations (17) and (18).
  (17) $$\begin{array}{l} v_{ij}^{\left(P\right)}=\eta \left(v_{ij}^{\left(P\right)},u_{ij}^{\left(P\right)}\right)+\epsilon_{\gamma }\left(\underset{m,n}{\sum }w_{ij}^{\left(P,mn\right)}+\underset{k}{\sum }w_{ij,k}^{\left(P,G\right)}\right) \end{array}$$
  (18) $$\begin{array}{l} v_{ij}^{\left(D\right)}=\eta \left(v_{ij}^{\left(D\right)},u_{ij}^{\left(D\right)}\right)+\epsilon_{\gamma }\left(\underset{m,n}{\sum }w_{ij}^{\left(D,mn\right)}+\underset{k}{\sum }w_{ij,k}^{\left(D,G\right)}\right) \end{array}$$
- **Step4**
  Similarly, the neurons which encode “D4” and “120 ticks” in *L*_4_ in pitch and duration subnetworks also exhibit gamma activities, these neurons are also updated by Equations (17) and (18). However, it is important to note that the excitation of neurons in the genre cluster can cause the activities in the composer and title cluster due to the trained feedforward connections. However, the activities of genre-neurons prompt the interneurons to fire, and ultimately inhibit the neurons in the composer and title cluster.

**Figure 8:**
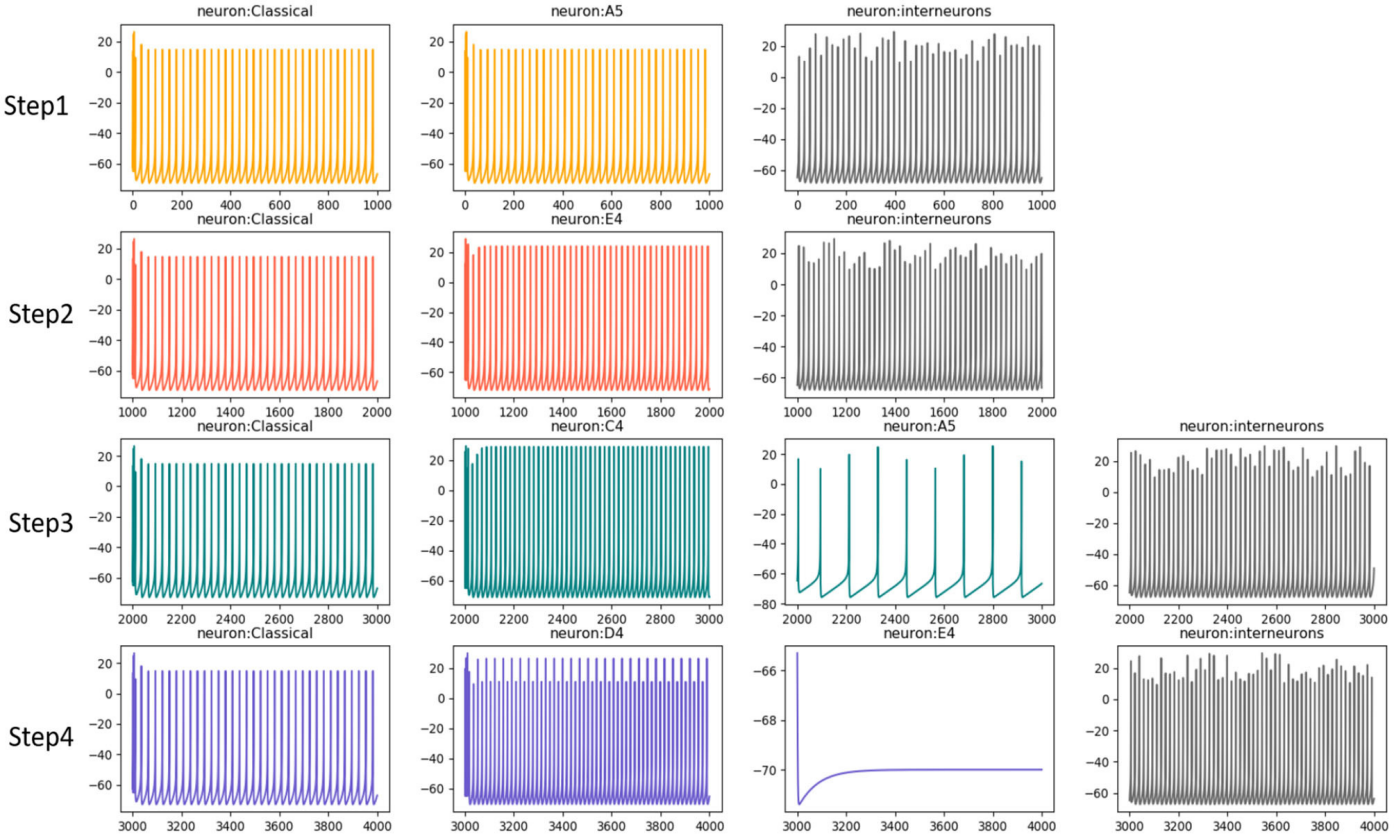
The neural oscillation during composition process. The neurons fire with gamma waves, however, the neurons *a*5 and *e*4 have slow or no activities since they fail in competitions.

Based on this process, Figure 8 draws the neurons' activities in each step mentioned above, they mainly exhibit gamma oscillations in this process. The neurons which prefer “c4” and “d4” in step3 and step4, respectively, are more active. However, the neurons that prefer “a5” and “e4” fail in the competition. Interneurons exhibit fast spiking (FS) pattern and also fire with gamma waves.

**Figure 9:**
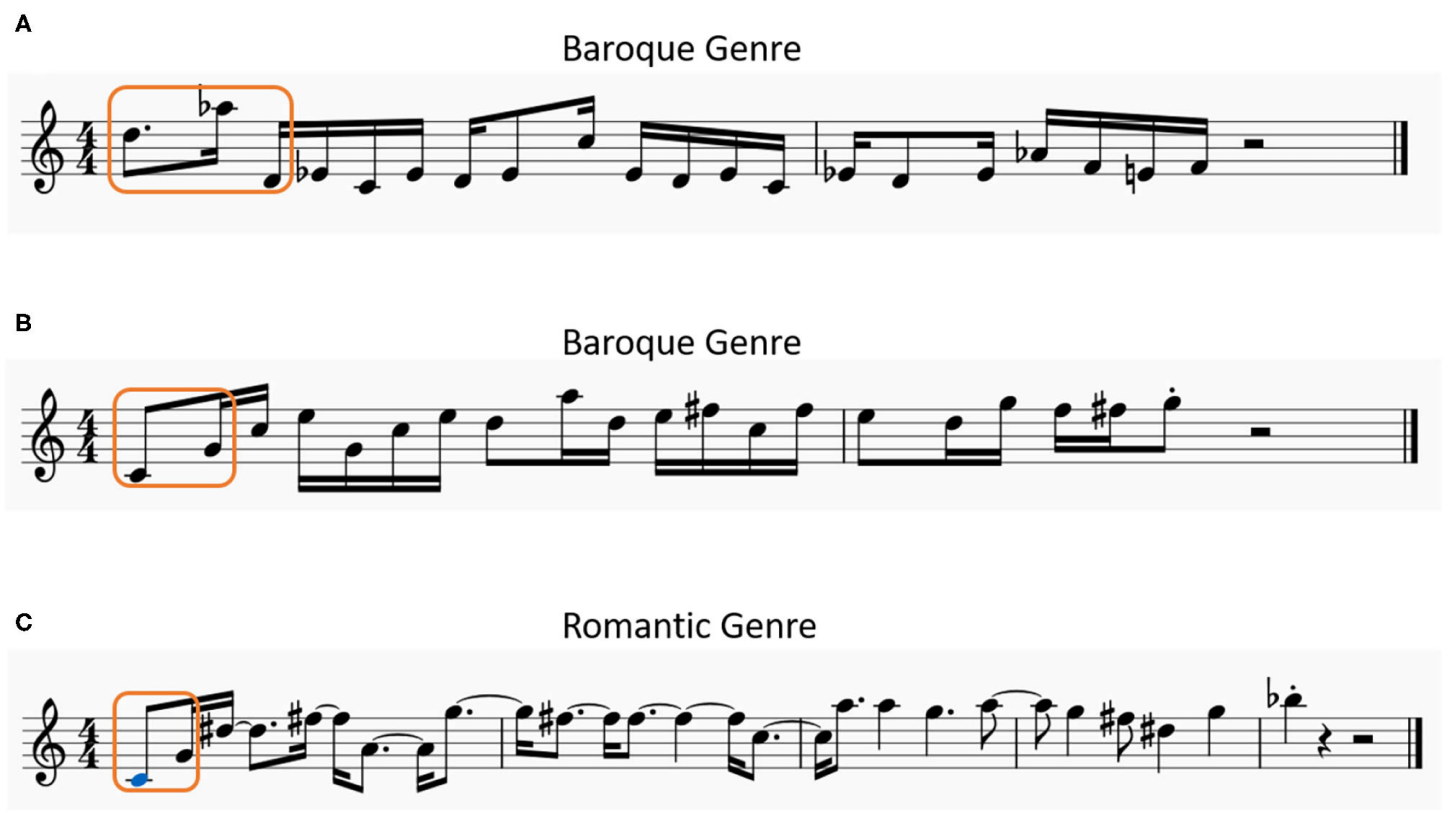
Three examples of melodies generated by the model, **(A,B)** have the same genre but begin with different seed notes, **(B,C)** are generated by the same seed notes but with the different genre characteristics.

Figure 9 shows three melodies generated by our model. Figures 9A,B are generated based on different seed notes with the baroque genre. Figure 9C is produced with the romantic genre but based on the same seed notes as Figure 9B.

#### 2.3.2. Composer-Based Composition

**Figure 10:**
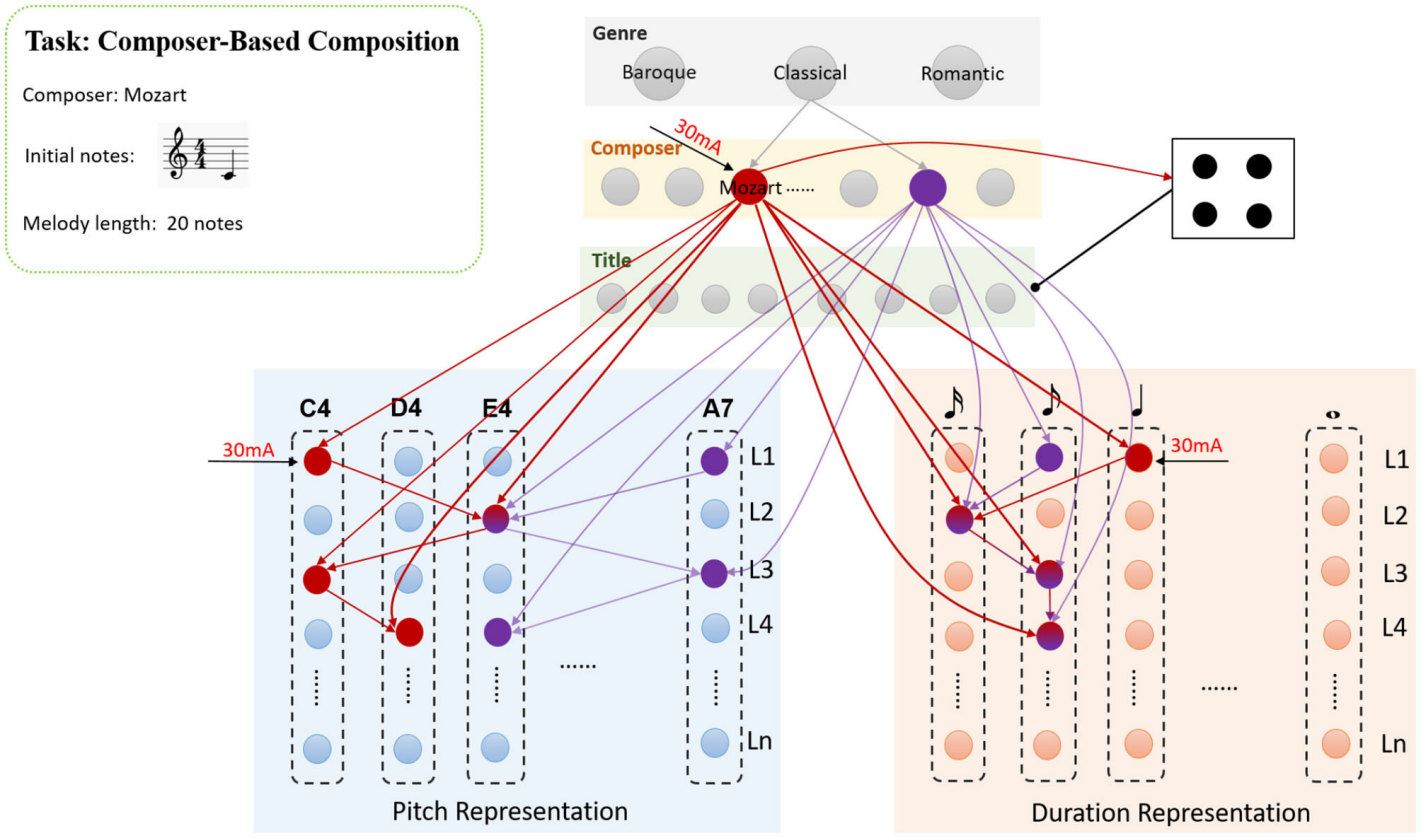
The process of the composer-based composition, a melody with Mozart's style is required to generate based on the composer cluster-interneurons-memory system circuits.

As is discussed in section 2.3, the styles of melodies can be very different due to the composers. This paper hopes to create melodies with composers' characters. Similarly, the neural circuits of composer cluster-interneurons-memory system are employed in this task. In order to explain the process clearly, an illustration is shown in Figure 10, the process actually is similar to the genre-based composition.

The task is to generate a Mozart style melody with 20 note-length, the seed note is *n*(*C*4, 480). Based on the task description, a 30mA current is injected into the neuron which represents the “Mozart” firstly, then the activities of this neuron make the interneurons inhibit the neurons in title cluster. The genre cluster keeps to be silent throughout the process. Since only one note *n*(*C*4, 480) is given at the beginning, the neurons (marked as red circles) in *L*_1_ of pitch and duration subnetworks receive the injected currents and fire with gamma frequencies. Then the trained synapses(red arrows) will trigger new neurons to fire orderly. The neurons in pitch and duration subnetworks are updated by Equations (19) and (20), which are similar to those in genre-based composition process.

(19) $$\begin{array}{l} v_{ij}^{\left(P\right)}=\eta \left(v_{ij}^{\left(P\right)},u_{ij}^{\left(P\right)}\right)+\epsilon_{\gamma }\left(\underset{m,n}{\sum }w_{ij}^{\left(P,mn\right)}+\underset{k}{\sum }w_{ij,k}^{\left(P,C\right)}\right) \end{array}$$

(20) $$\begin{array}{l} v_{ij}^{\left(D\right)}=\eta \left(v_{ij}^{\left(D\right)},u_{ij}^{\left(D\right)}\right)+\epsilon_{\gamma }\left(\underset{m,n}{\sum }w_{ij}^{\left(D,mn\right)}+\underset{k}{\sum }w_{ij,k}^{\left(D,C\right)}\right) \end{array}$$

**Figure 11:**
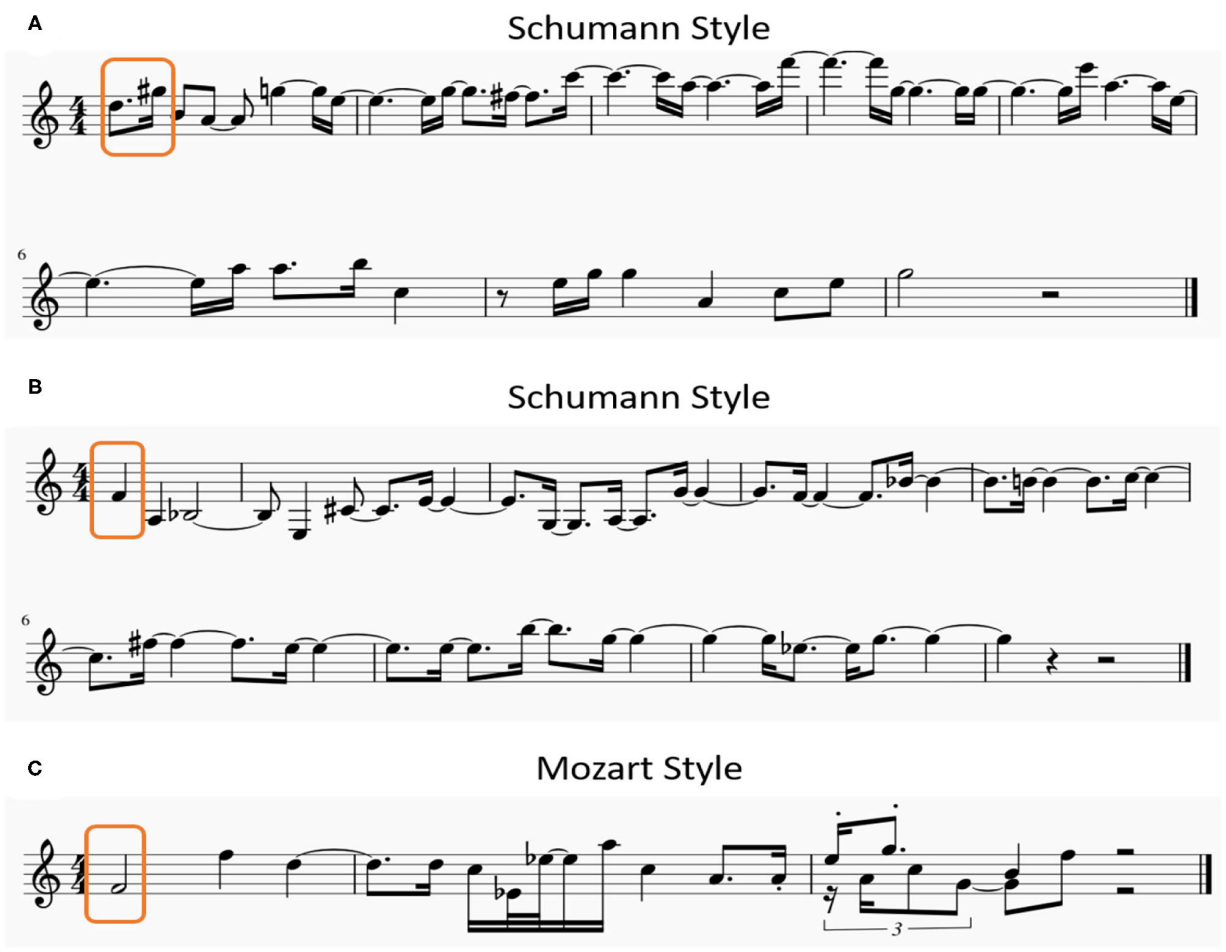
Three examples of melodies generated by the model, **(A,B)** have the same composer's style but begin with different seed notes, **(B,C)** are generated by the same seed notes but with the different composers' characteristics.

These formulas demonstrate that, besides the input from their adjacent neurons in the same subnetwork, these neurons mainly receive the input from the composer cluster rather than the genre cluster. The following notes generated by the model are *n*(*E*4, 120), *n*(*C*4, 240), *n*(*D*4, 240), and etc. Only a part of the generated notes are shown in this figure for simplicity, the model can generate a melody with 20 note-length according to the requirements of the task. Similarly, three generated melodies based on different seed notes with different composers' characteristics are illustrated in Figure 11.

Figures 8, 11 only show the samples created by our model. More generated melodies can be found in the Supplementary Material.

## 3. Results

### 3.1. Dataset

We use a public corpus (Krueger, 2018), which provides 331 classical pieces from 25 famous musicians recorded by MIDI format for training our model. MIDI standard is a world protocol that connects digital musical instruments, computers, tablets and smartphones. A musical piece can be recorded as symbolic patterns in MIDI format. The musicians and their genres are summarized in Table 2. The genre is divided to baroque, classical and romantic genres. In fact, there are more detailed divisions of the genre in the romanticism period, however, these genre branches are not considered for the time being in article.

**Table 2:** The basic information of musicians and their genres.

**Genre**	**Musician**
Baroque	Bach
Classical	Haydn, Mozart, Beethoven, Schubert, Clementi,
Romantic	Mendelssohn, Liszt, Chopin, Schumann, Brahms, Burgmueller, Debussy, Godowsky, Moszkowski, Mussorgsky, Rachmaninov, Ravel, Tchaikovsky, Albéniz, Balakirew, Borodin, Granados, Grieg, Sinding

### 3.2. Encoding Experiments

**Figure 12:**
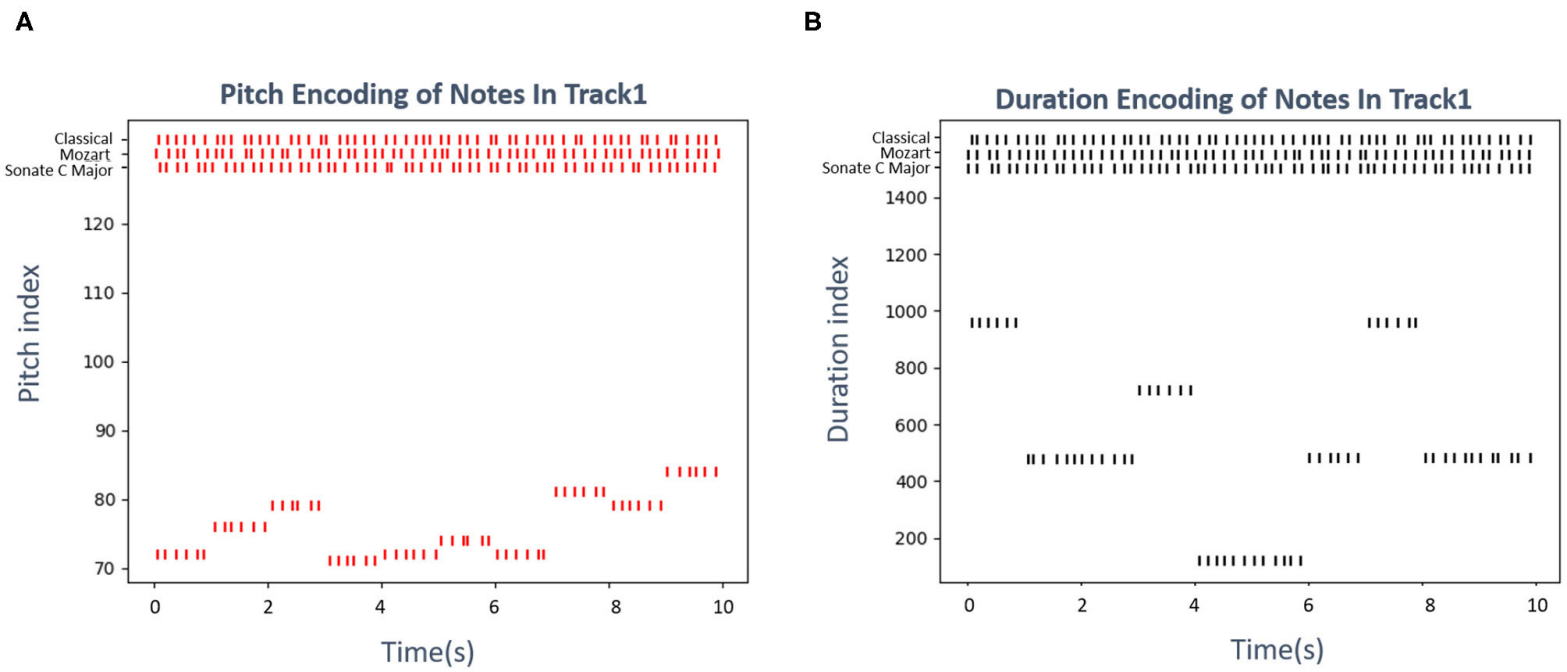
The encoding results of the Mozart's famous work “Sonate C Major, K545.” **(A)** Shows the firing results of neurons in pitch subnetwork, the vertival axis presents the pitch index that the neuron prefers, the horizontal axis presents the time. **(B)** Exhibits the activities of neurons in duration subnetwork, the vertical axis is the duration(ticks defined in MIDI standard) which the neuron prefers, the horizontal axis indicates the time.

Musical pitches and durations have been defined as 0 127 digits and several ticks, respectively, by MIDI standards. For example, the pitch “c5” is represented as 72, a crotchet always lasts 480 ticks. This paper employs a famous classical piano work, “Sonate C Major, K545” written by Mozart as an illustration. Figures 12A,B shows the encoding results of pitch and duration subnetwork, respectively, as the time passes, neurons preferred the notes of this piece emit spikes orderly. The neurons which prefer the “Classical,” “Mozart,” and “Sonate C Major” in knowledge subnetwork continue to fire throughout the whole process. This graph only shows the results of 10 notes in track 1 of this example for simplicity.

### 3.3. Composition Experiments

To evaluate the quality of the generated melodies, 41 human listeners, including 19 males and 22 females, are involved to complete a user study. Five (all females) of them majored in piano or composition, and the rest had no music experience. We considered those five people who have music backgrounds as professionals for short. Our experiments are based on these two groups of subjects, professional and non-professional groups. We design three experiments to evaluate the melodies generated by our model, the details are discussed in the following sections. Actually, the capacity of the memory system needs to be measured, the details and results can be found in our previous work (Liang et al., 2020), this paper focuses on the issue of melody composition.

#### 3.3.1. Evaluation on the Composition Quality

**Figure 13:**
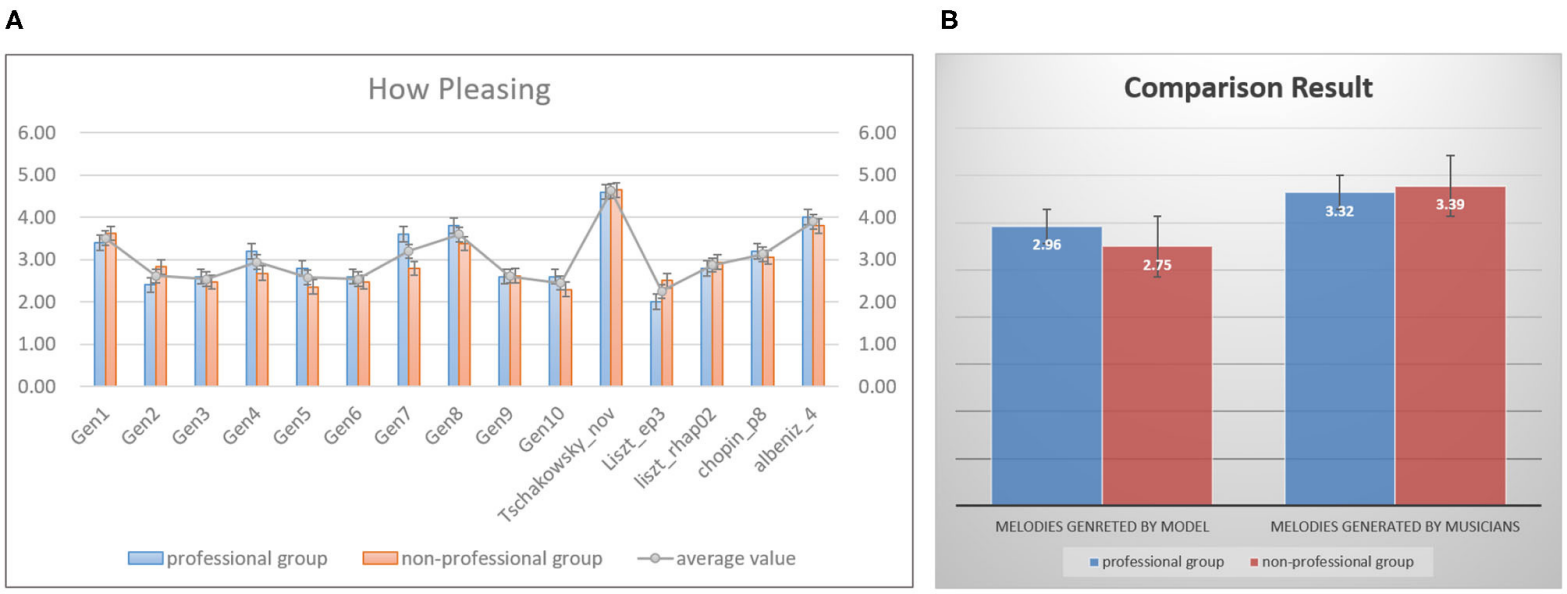
The statistical results of a user study, for people (blue bars) with musical backgrounds and (orange bars) without musical backgrounds, **(A)** shows the average value of each melody in the testset, panel **(B)** exhibits that the total mean value of melodies generated by the model and musicians.

The purpose of the first experiment is to evaluate whether the melodies produced by our model are pleasant to hear. In this task, we randomly collected 15 melodies as a testset, ten of which were generated by our model, the rest were extracted from the melodies written by musicians in our dataset. Subjects were asked to listen to these 15 melodies and score them, and they were not told about the testset contains the pieces created by musicians. The score ranges from 1 to 5, 1 means bad, 5 means very pleasing. Figure 13A shows the statistical result of each melody. The blue bars represent the average value graded by the professional group, the orange bars express those from the non-professional group. The gray line calculates the mean value of each melody based on the scores from two groups. The first ten melodies (marked as “Gen1” to “Gen10”) are generated by our model, and the rest melodies are extracted from the works written by Liszt, Chopin and etc. We have found that the results from professional and non-professional groups are basically consistent. The score 4.62 of melody coming from Tschaikovsky is the highest one since this melody is really pleasing, however the one coming from the work “Liszt_ep3” only obtain the low score 2.25. The generated melodies, “Gen1”(3.51), “Gen4”(2.94), “Gen7”(3.2), and “Gen8”(3.59) sound nice, the scores of these four melodies are even higher than those written by musicians. Figure 13B shows the total average results of melodies created by the model and musicians. Totally, musicians write better melodies, our model can write several nice pieces. Furthermore, it is interesting that the professional group gives a higher score than the non-professional group about the generated melodies. Through the feedback from the subjects, we conclude the possible reasons as follows, (1) Most subjects in non-professional groups have never heard classical music, they really do not like classical music. Hence they give the lower scores. (2) Subjects in the professional group know a lot about classical music, they argue that the melodies generated by the model are not much worse than those produced by humans.

#### 3.3.2. Evaluation on Genre-Based Composition

**Figure 14:**
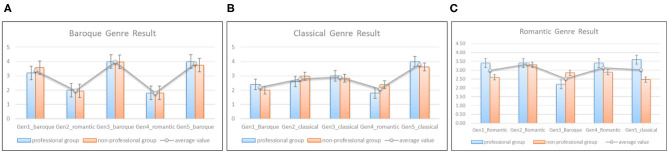
The statistical results of genre-based composition, **(A)** shows the results given by professional (blue bars) and non-professional (orange bars) groups on whether the melody have the characteristics of Baroque period, **(B,C)** exhibit the results of classical and romantic periods.

The second task is to evaluate whether the styles of generated melodies are similar to genre characteristics. The professional and non-professional groups still participate in this experiment. Since the dataset involves baroque, classical and romantic three genres, we randomly select five melodies generated by the model as a testset for each genre, respectively. We mixed two melodies which belong to other genres into the genre testset as the noises. The participants were asked to listen to five representative works of each genre, and then listen to the corresponding testset. To avoid bias, the subjects also did not know the details of each testset. The score also ranges from 1 to 5, however, 1 means the melody has no genre features, 5 means that the melody style is quite similar to the genre. The average values of generated melodies are summarized in Figure 14. The blue and orange bars represent the mean values graded by the professional and non-professional groups of each melody, respectively. Figure 14A shows that the melodies generated with baroque style got a higher score than the other two noise ones. It demonstrates that our model can produce melodies with baroque style well. Similarly, Figures 14B,C shows that the melodies with classical and romantic styles also got a high score compared with noisy melodies. However, the melody “Gen1_romantic” and “Gen5_classical” in Figure 14C nearly got the same score(3.03 and 3, respectively). It means that the style of “Gen5_classical” is ambiguous. In general, the result of genre-based melody composition is inspiring.

#### 3.3.3. Evaluation on Composer-Based Composition

**Figure 15:**
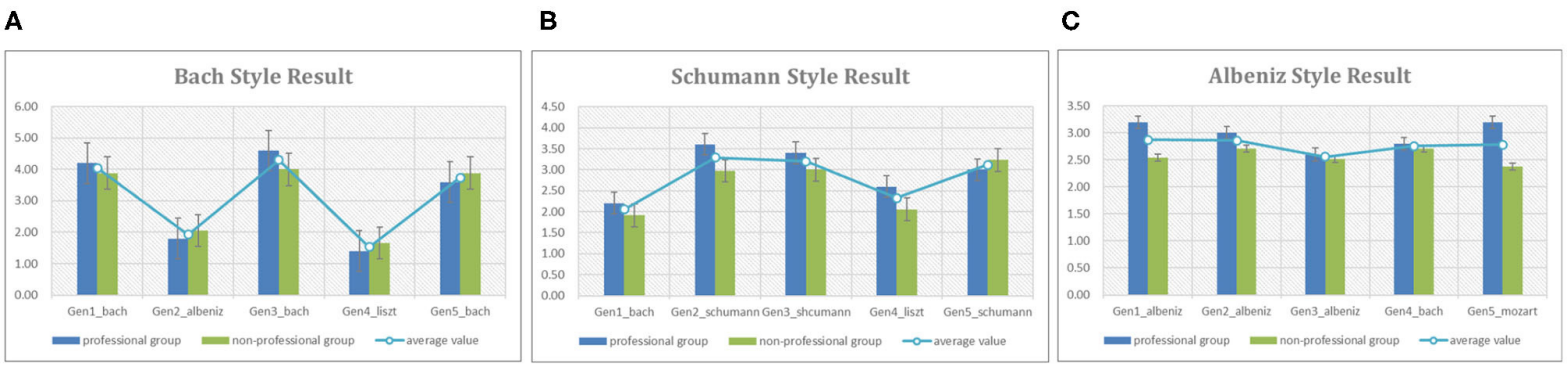
The statistical results of composer-based composition, **(A)** shows the results given by professional (blue bars) and non-professional (green bars) groups on whether the melody have the Bach's style, **(B,C)** exhibit the results of Schumann and Albeniz.

Similar to the section 3.3.2, this experiment is to evaluate whether the generated melodies have the composers' styles. We randomly picked 3 out of 25 composers as the targets, and collected 5 generated melodies as a testset for each target composer. We also mixed 2 melodies generated by our model with other composers' styles into the testset as the noises. Figure 15 shows the total results of this task. In this figure, Bach, Schumann, and Albeniz are picked as target composers, the blue and green bars describe the average values of each test melody by professional and non-professional groups. Figures 15A,B indicate that the scores of melodies with Bach and Schumann's styles generated by the model are significantly higher than the noisy melodies. However, Figure 15C shows that the melody named “Gen3_albeniz” got the lowest score, even lower than the noisy melodies. Actually, the differences of melodies in Albeniz's group are not remarkable, “Gen1_albeniz” and “Gen2_albeniz” got 2.87 and 2.86, while the scores of “Gen4_bach” and “Gen5_mozart” are 2.76 and 2.79. We discussed with the participants, the possible reasons may be that the melodies of Albeniz have not significant characteristics because of no accompaniments, the generated melodies have not remarkable styles either. Furthermore, the participants have their own preferences. They say their assessments are indeed very subjective.

## 4. Discussion

This paper introduces a brain-inspired spiking neural network to learn and create the musical melodies with different styles. Based on the brain mechanisms, we build two subsystem, the knowledge and memory subsystems, to achieve our goals. A hierarchical structure is utilized to learn and store the basic information of a musical piece. The genre, composer and title cluster encode and memorize the corresponding information of a piece. Besides, interneurons are involved in this system to perform the composition task. Sequential memory system encodes and stores the ordered musical notes. All the neurons are simulated by the Izhikevich model, both regular and fast spiking patterns are used in our model. During the learning process, synapses between neurons are updated by the STDP learning rule. Genre-based and composer-based melody composition can be achieved depending on the different circuits, different neural clusters are activated in these tasks. The experiments shows that our model can generate melodies with different styles of genres and composers. Some of them sound nice and have strong characteristics.

Based on the experiments mentioned in section 3, the model runs on the supercomputer. In fact, the scale of the network is increasing with the number of the input musical pieces, the network scale and storing capacity have been discussed in our previous work (Liang et al., 2020). Since the algorithm is not parallel, the model has no special requirement for CPU, but it requires 22 GB of memory. The learning process needs about 50 h. However, the time cost of the composition process depends on the length of generated melodies. In our experiment, the shortest and the longest melodies contain 20 and 50 notes, the time cost of generating one melody ranges between 3 and 20 min.

To our best of knowledge, this work is the first attempt to create musical melodies using a spiking neural network based on neuroscientific findings. There are many hypotheses in our method since the corresponding brain mechanisms are not clear. The experiments are inspiring, however, there are many issues needed to be discussed further.

- The knowledge learning problem in this paper needs to be developed further. Knowledge involves the semantic information, it involves multi-modal concepts in brain. The representation and learning problems are needed to be explored deeply in the future work.
- The melody generation in this paper mainly depends on the sequential memory system, this process is similar to musical improvisation. However, the creation of a beautiful musical piece needs more musical theories, such as chords, harmony, rhythms, and etc. The feedback from people who have musical backgrounds indicated that the rhythms of our generated melodies are not steady. Actually, this is our key task in the next paper.
- This paper focuses on the melody composition. However, a music piece has more than one part or voice. How to generate accompaniments or four part harmony is our important work in future.
- Actually, to compare our method with the existed models, we have investigated many methods achieved by traditional artificial neural networks (ANNs). We have found that the task of our model is very different from those achieved by the existed methods. First, we mainly generate the melodies, this task can be called composition in musical theory, while many tasks achieved by traditional methods always have accompaniments. The comparison is not fair. Second, our task is based on classical music, some tasks using ANNs focus on Jazz, blues, pop music, or other musical genres. The comparison is still difficult. However, the model needs to be improved deeply in our future work, including generating accompaniments, learning more musical styles and etc.
- A more perfect evaluation system is a big challenge for this topic. Actually, people who took part in our experiments have told us that their assessments were very subjective, quite a few of them have no idea about classical music. How to explore a more reasonable and effective method is very important in our future work.

## Data Availability Statement

The original contributions presented in the study are included in the article/Supplementary Material, further inquiries can be directed to the corresponding author/s.

## Author Contributions

QL and YZ proposed the idea, formulate the problem, performed the experiments, analyzed the results, and wrote the paper. QL designed and developed the algorithm. Both authors contributed to the article and approved the submitted version.

## Conflict of Interest

The authors declare that the research was conducted in the absence of any commercial or financial relationships that could be construed as a potential conflict of interest.

## Supplementary Material

The generated melodies are available at https://bii.ia.ac.cn/ai-music/.
