# Supplementary material for: Stylistic Composition of Melodies Based on a Brain-Inspired Spiking Neural Network
Source: Front Syst Neurosci. 2021 Mar 11;15:639484. doi: 10.3389/fnsys.2021.639484 (PMC7991719; doi:10.3389/fnsys.2021.639484)
Supplement: Supplementary file 1 [file Data_Sheet_1.PDF]

Supplementary Material: Stylistic composition of melodies based on a brain-inspired spiking neural network

1 SUPPLEMENTARY DATA

We proposed a spiking neural network inspired by brain mechanisms to create musical melodies with different styles. As is shown in Fig.S1, the training set is a collection of MIDI files, which record classical piano works from different musicians. After the learning process, the model can generate melodies and store them as MIDI files. To better understand the melodies generated by our model, we list 20 generated samples picked randomly in our testset which are shown in Fig.S2.

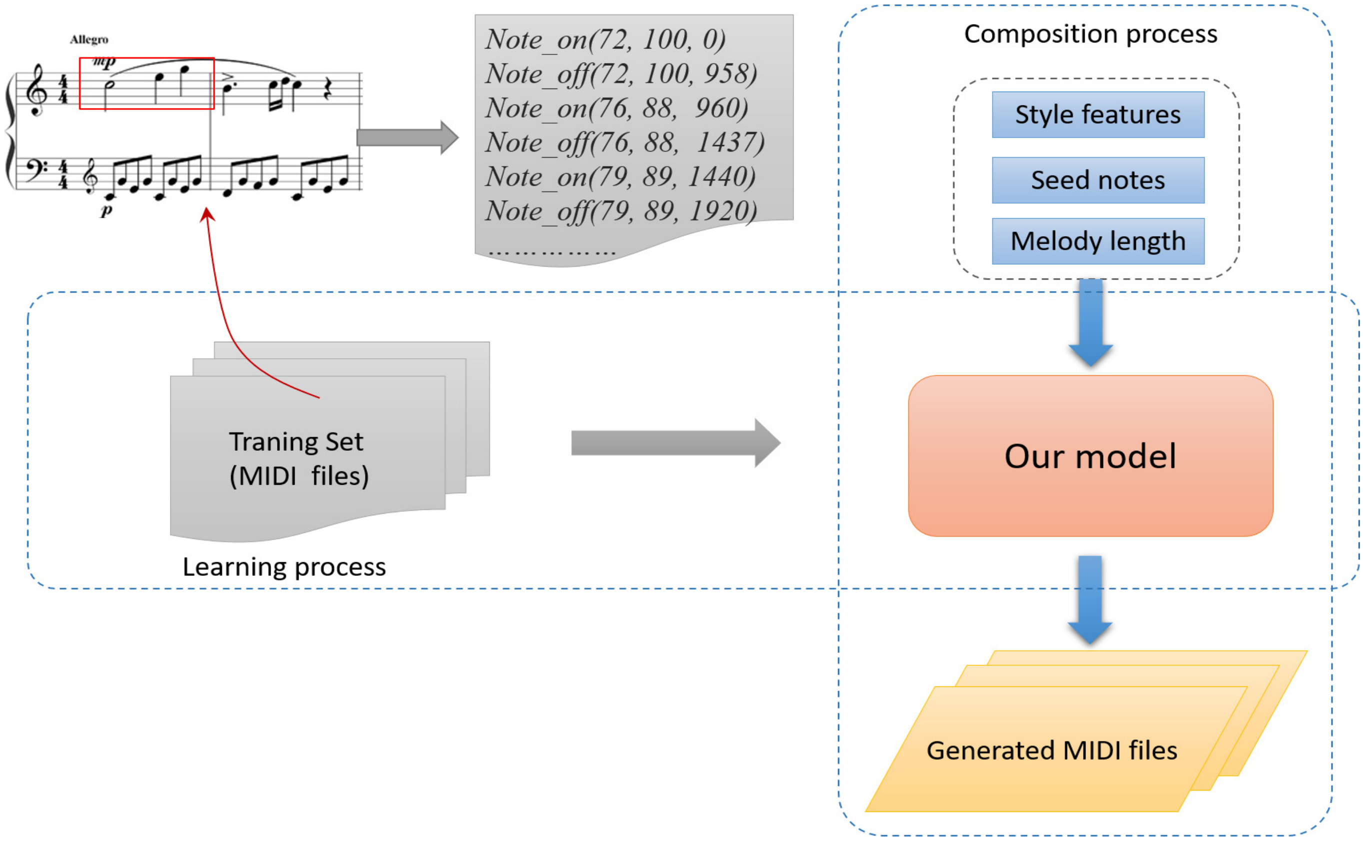

Figure S1. The training set is composed of numerous MIDI files, all the musical information is stored as MIDI events. The model learns these MIDI files and generated melodies which are also written by MIDI files.

(1)

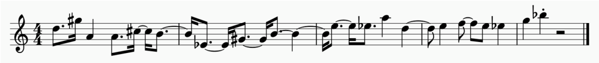

(2)

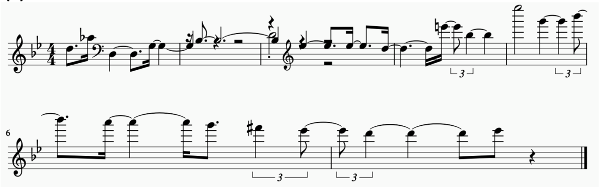

(3)

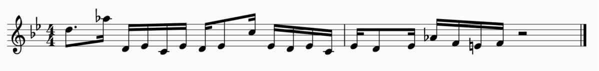

(4)

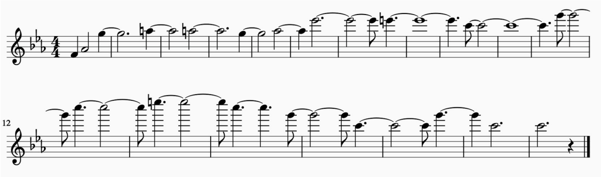

(5)

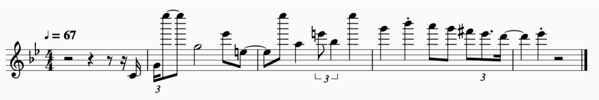

(6)

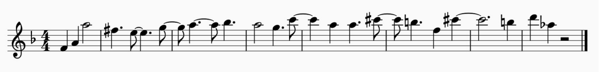

(7)

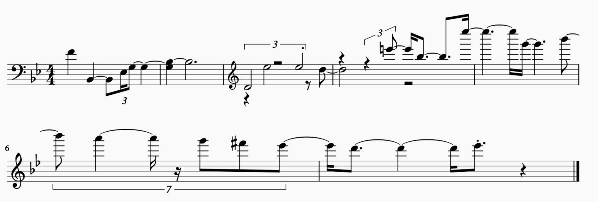

(8)

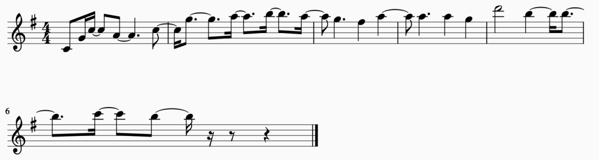

(9)

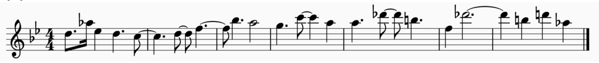

(10)

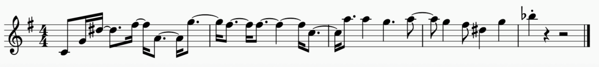

(11)

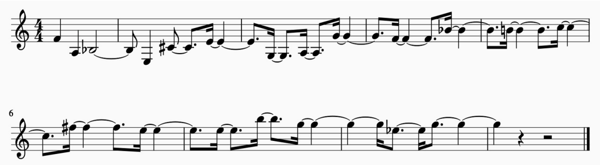

(12)

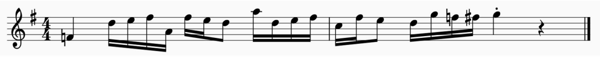

(13)

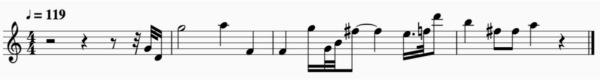

(14)

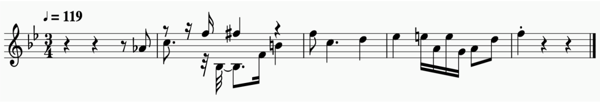

(15)

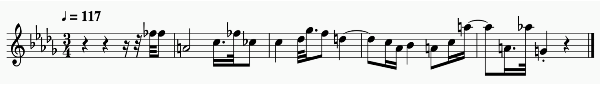

(16)

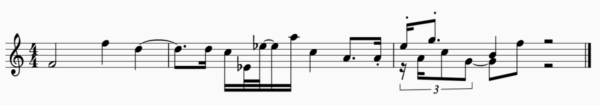

(17)

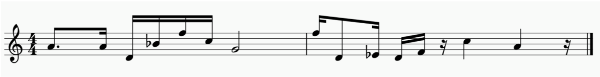

(18)

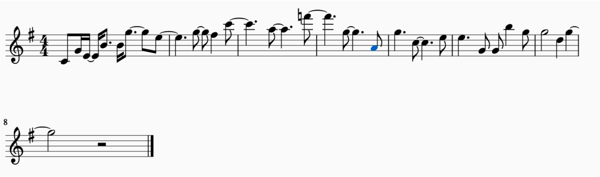

(19)

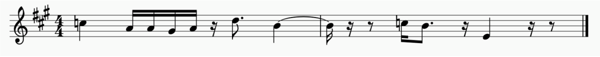

(19)

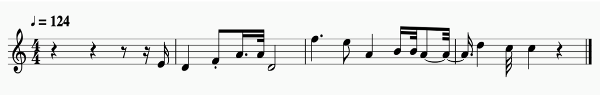

(20)

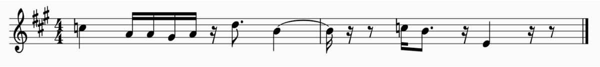

Figure S2. 20 randomly selected melodies which are generated by our model.
